# A Difference-in-Differences Approach to Assess the Effect of a Heat Action Plan on Heat-Related Mortality, and Differences in Effectiveness According to Sex, Age, and Socioeconomic Status (Montreal, Quebec)

**DOI:** 10.1289/EHP203

**Published:** 2016-05-20

**Authors:** Tarik Benmarhnia, Zinzi Bailey, David Kaiser, Nathalie Auger, Nicholas King, Jay S. Kaufman

**Affiliations:** 1 Institute for Health and Social Policy, McGill University, Montreal, Quebec, Canada; 2 Direction de santé publique de l’Agence de la santé et des services sociaux de Montréal, Montréal, Québec, Canada; 3 Institut National de Santé Publique du Québec, Montréal, Québec, Canada; 4 Biomedical Ethics Unit, and; 5 Department of Epidemiology, Biostatistics, and Occupational Health, McGill University, Montreal, Quebec, Canada

## Abstract

**Background:**

The impact of heat waves on mortality and health inequalities is well documented. Very few studies have assessed the effectiveness of heat action plans (HAPs) on health, and none has used quasi-experimental methods to estimate causal effects of such programs.

**Objectives:**

We developed a quasi-experimental method to estimate the causal effects associated with HAPs that allows the identification of heterogeneity across subpopulations, and to apply this method specifically to the case of the Montreal (Quebec, Canada) HAP.

**Methods:**

A difference-in-differences approach was undertaken using Montreal death registry data for the summers of 2000–2007 to assess the effectiveness of the Montreal HAP, implemented in 2004, on mortality. To study equity in the effect of HAP implementation, we assessed whether the program effects were heterogeneous across sex (male vs. female), age (≥ 65 years vs. < 65 years), and neighborhood education levels (first vs. third tertile). We conducted sensitivity analyses to assess the validity of the estimated causal effect of the HAP program.

**Results:**

We found evidence that the HAP contributed to reducing mortality on hot days, and that the mortality reduction attributable to the program was greater for elderly people and people living in low-education neighborhoods.

**Conclusion:**

These findings show promise for programs aimed at reducing the impact of extreme temperatures and health inequities. We propose a new quasi-experimental approach that can be easily applied to evaluate the impact of any program or intervention triggered when daily thresholds are reached.

**Citation:**

Benmarhnia T, Bailey Z, Kaiser D, Auger N, King N, Kaufman J. 2016. A difference-in-differences approach to assess the effect of a heat action plan on heat-related mortality, and differences in effectiveness according to sex, age, and socioeconomic status (Montreal, Quebec). Environ Health Perspect 124:1694–1699; http://dx.doi.org/10.1289/EHP203

## Introduction

Heat waves are a public health concern that is likely to grow in importance in the context of climate change (Huang et al. 2012; Li et al. 2013). The impacts of heat waves on mortality have been extensively documented (Åström et al. 2011; Basagaña et al. 2011; Basu 2009; Borrell et al. 2006; Bridger et al. 1976; Ellis 1972; Jones et al. 1982; Rooney et al. 1998). The 1995 Chicago, Illinois, heat wave resulted in 700 excess deaths over only a few days (Semenza et al. 1996). In Europe, around 15,000 excess deaths were reported during a heat wave in the first week of August 2003 (Robine et al. 2008). More recently, approximately 55,000 excess deaths (including potential contributing effects of wildfire smoke) were associated with the 2010 heat wave in Russia (Barriopedro et al. 2011). In response to such events, public health authorities have developed programs aimed at reducing heat wave-related health effects, including heat action plans (HAPs).

HAPs include early alerts and advisories combined with emergency public health measures to reduce heat-related morbidity and mortality (Kovats and Hajat 2008; Kovats and Kristie 2006; Lowe et al. 2011; McGregor et al. 2015). HAPs are activated when meteorological conditions meet local criteria for classification of hot days, each jurisdiction having its own precise criteria (Tong et al. 2010). Variations exist in terminology to describe such public health programs (Toloo et al. 2013). In this paper, we use the term “HAPs” to refer to such programs.

The content of HAPs is generally based on existing empirical evidence of risk and protective factors for heat-related health impacts. In the absence of evaluations of actual HAP implementation, however, it remains unknown whether the various activities carried out in a HAP have an effect on heat-related mortality and morbidity. Such evidence would help inform future interventions aimed at reducing heat-related health impacts. Evaluating the effectiveness of public health programs is essential for several reasons: *a*) to assess whether policies effectively prevent and control mortality and morbidity; *b*) to evaluate whether programs are ethical, a good use of public resources, and efficient in reducing social inequalities; and *c*) to determine whether programs should be renewed or improved at the end of an initial trial period.

Very few studies have assessed the effectiveness of HAPs on health. In two recent reviews (Boeckmann and Rohn 2014; Toloo et al. 2013), only seven studies worldwide assessed the effectiveness of HAPs in reducing mortality. Six studies found that fewer people died of heat-related complications after the implementation of a HAP than was expected had the program not been in place (Chau et al. 2009; Ebi et al. 2004; Fouillet et al. 2008; Palecki et al. 2001; Tan et al. 2007; Weisskopf et al. 2002), whereas one study did not report an association (Morabito et al. 2012). Some of these studies were descriptive, using a pre–post approach to estimate the reduction in mortality attributable to the HAP by comparing the number of deaths on hot days before HAP implementation with the number after implementation. A pre–post approach may document variation in mortality, but, because it does not account for potential confounding (e.g., factors other than HAPs that are associated with heat-related mortality and have changed over time), does not provide evidence of causal effects associated with the program, which is fundamental to formulating and implementing effective HAPs. Other studies (Ebi et al. 2004; Morabito et al. 2012) used control groups (e.g., other cities) and controlled for measured confounders, but still may have been biased by unmeasured confounders. To deal with unmeasured confounders, quasi-experimental methods have been developed for observational studies as alternatives to experimental methods and are used to provide estimates from observational studies. The term “quasi-experiment” refers to the analysis of an observed treatment in relation to an outcome measure in which assignment to the treatment is not made by physical randomization, as in a true experiment, but is instead assigned according to some mechanism that can be argued to be independent of the potential outcome of each experimental unit. Using the counterfactual framework, natural experiments can be used to estimate the causal effect of specific policies on population health.

The European heat wave of 2003 prompted the Montreal Public Health Department (PHD) to develop a HAP to reduce heat-related mortality and morbidity (Price et al. 2013). Implemented in 2004, the HAP comprises a spectrum of interventions triggered at different alert levels based on temperature forecasts of Environment Canada. Important interventions undertaken as part of the HAP by the Montreal PHD and partners are triggered at the alert level (called “Active watch” level in the Montreal HAP), when daily temperatures exceed 30°C (Price et al. 2013). These interventions include, for example, public advisories via different media about preventive measures, and intensified surveillance and implementation of preventive measures in health care facilities (Appendix 1). The alert level is followed by two other levels, associated with additional interventions such as extended operational hours of public pools and opening of air-conditioned shelters. The HAP is active on days defined as hot, and not active on other summer days. The program is automatically triggered by meteorological conditions, and targets the entire population of the Island of Montreal. Although implemented in 2004, mortality attributable to heat continues to be observed in Montreal (Benmarhnia et al. 2014b), with 105 heat-related deaths occurring during a 5-day heat wave in 2010 (Price et al. 2013) suggesting the need to evaluate this program. Evidence about the effectiveness of the HAP is essential to improve this program.

The impact of heat waves on mortality is uneven, and economically or socially disadvantaged populations are at higher risk for heat-related death. Potential factors associated with vulnerability to heat-related mortality, including age, sex, education, and neighborhood socioeconomic status (SES), have been documented (Benmarhnia et al. 2015a; Gronlund 2014). It is thus worthwhile to assess whether HAPs are effective in reducing or eliminating unequal health outcomes associated with heat. However, an assessment of the heterogeneity of potential benefits across subpopulations or territories (e.g., neighborhoods) is lacking in the policy evaluation literature (Bauman et al. 2014; Benmarhnia et al. 2014a).

The equity impact of a policy, defined as the differential reduction of mortality between vulnerable and nonvulnerable populations, can be assessed by using a quasi-experimental method to identify heterogeneity in potential benefits among subpopulations or territories. We define as “equitable” any policy that reduces inequality in which vulnerable populations start from a comparatively worse-off position. For example, a policy that reduces inequalities in mortality between the elderly and other age groups (e.g., daily mortality differences between the two age groups) is by our definition equitable, if the elderly experienced higher mortality rates before its implementation. We stress, however, that one can employ alternative definitions of equity when using our method.

Using a difference-in-differences approach (a quasi-experimental method that can be used to estimate the causal effects of public health policies) (Angrist and Pischke 2008; Basu et al. 2016), we aimed to estimate the causal effect of the HAP program on mortality in Montreal. We assessed whether the HAP program had a heterogeneous effect across sex, age, and neighborhood socioeconomic status (SES).

## Methods

### Data Sources

The study population included all residents of the Island of Montreal who died during the months of June, July, and August between 2000 and 2007, inclusively, and were listed in the provincial death registry. We restricted the population to non-accidental causes of death, excluding deaths for *International Classification of Diseases, 9th Revision* (ICD-9) codes 800–999 (injury and poisoning) and *10th Revision* (ICD-10) codes S00–T98 (injury, poisoning, and other consequences of external causes). Individual information on age, sex, date of death, and census tract of residence was available for each death. Deaths were aggregated daily. We used the percentage of residents > 20 years of age without a high school diploma in the census tract of residence (at time of death) to assign a “neighborhood SES” to each participant in the study; data were extracted from the 2006 census (Benmarhnia et al. 2015b). We generated three strata of neighborhood SES by dividing the data into tertiles, and compared the first and third tertiles in the analysis.

We obtained maximum outdoor temperatures for the months of June through August between 2000 and 2007 from the Environment Canada meteorological observation station at the Montreal Pierre Elliott Trudeau International Airport (Environment Canada 2015). The meteorological observation station used in this study is also used for activation of the Montreal HAP.

### Study Design and Statistical Analysis

We used a difference-in-differences (DID) design, in which daily mortality for the population of Montreal was compared before and after HAP implementation in 2004. Summer days (June–August) were classified as “eligible” if they were hot enough to have triggered an “active watch” alert level according to the HAP (i.e., if they would have been eligible for a HAP intervention), or as “non-eligible” otherwise, regardless of whether they occurred during 2000–2003 (before the HAP was implemented) or during 2004–2007 (after the HAP was implemented). The counterfactual quantity of interest is the difference in the daily number of deaths between eligible (hot) and non-eligible (non-hot) days that would have occurred during 2004–2007 if the HAP had not been implemented. Therefore, the causal effect of the HAP on daily mortality was estimated as the difference between two values: *a*) the difference in number of deaths on eligible (hot) days before and after implementation of the HAP, and *b*) the difference in number of deaths on ineligible (non-hot) days before and after implementation of the HAP. We thus obtained a DID estimate to represent the causal effect of the HAP program on daily mortality in Montreal.

The DID approach will generate a valid estimate of the causal effect if the implementation of the HAP was the only factor that might cause a change in the association between heat and mortality before and after HAP implementation. We chose a short interval of time (4 years before and 4 years after initiation of HAP), to limit potential confounding due to population acclimatization and urban infrastructure changes (Petkova et al. 2014) that might reduce the effect of hot days on mortality during the post-HAP time period. We also compared the distributions (using a Kolmogorov–Smirnov test for equality of distribution functions) of maximum daily temperatures on eligible and non-eligible days during each time period to determine whether, for example, higher maximum daily temperatures on eligible days during the post-HAP time period might bias our estimate of the effect of the HAP by increasing mortality on eligible days.

We estimated the HAP effect on daily mortality using a time series analysis in DID Poisson models adjusted to account for temporal patterns (month, week, and day). To calculate the daily number of deaths “prevented” during hot days after HAP implementation, we predicted the Poisson count from the adjusted models using the DID estimate. The DID estimate represents the product interaction term between the estimated effect of eligible (hot) versus non-eligible (non-hot) days and the estimated effect of the pre-HAP (2000–2003) versus post-HAP (2004–2007) time period. A positive value of the DID estimate generated with this method represents an estimated reduction of daily mortality attributable to the HAP, because we compared pre-HAP deaths to post-HAP deaths. We estimated 95% confidence intervals (CIs) for the adjusted DID estimate by bootstrapping (1,000 samples) (Carpenter and Bithell 2000).

As sensitivity analyses, we tested the validity of the causal model by conducting the same analyses above, but defining HAP at arbitrary implementation periods: *a*) in 2000 and *b*) in 2002. We compared differences in mortality on eligible and non-eligible days between 4-year time periods that were unrelated to the HAP intervention: *a*) 1996–1999 versus 2000–2003; and *b*) 1998–2001 versus 2002–2005. We then tested different definitions of hot days (thus modifying the assignment of “eligible” and “non-eligible” days) in order to test the validity of the causal model. We also used a definition of hot days as maximum temperatures above 28°C and above 32°C, expecting no effect of the HAP on mortality reduction. We considered the cumulative heat effect (lag 0–5: up to 5 consecutive hot days) (Åström et al. 2011), expecting a larger effect of the HAP on mortality reduction by considering consecutive hot days. We selected this lag period based on previous studies in Montreal (Goldberg et al. 2011). We also calculated the DID estimate considering a harvesting effect. This is the hypothesis that some frail individuals who died during a hot day would have died in the subsequent days or weeks even without the hot day. These deaths are strictly speaking not caused by the hot day, but only hastened by it. To estimate this effect, we calculated a displacement ratio after 15 days (Saha et al. 2014). We then multiplied the displacement ratio we obtained with the DID estimate to get a DID estimate that takes into account the harvesting effect. Finally, we calculated the DID estimate by restricting “non-eligible” days to maximum temperatures between 25°C and 29°C.

### Equity of Program Impact

To study equity in implementation, we assessed whether the HAP had a heterogeneous effect by sex, age, and neighborhood SES. We first dichotomized each of the variables: *a*) sex: men vs. women; *b*) age (≥ 65 years vs. < 65 years); *c*) SES: first versus third tertile of neighborhood education. We then calculated daily mortality differences between each of the two categories (e.g., daily mortality among men minus daily mortality among women). The HAP effect on daily mortality differences was estimated following the same method above. Thus, to assess heterogeneity in the program effect, we calculated differences-in-differences-in-differences (DIDID) estimates using a DID estimate (i.e., interaction term) as above, but considering as the health outcome a daily difference between two groups.

## Results

### Descriptive Statistics

During the study period (2000–2007), 28,137 non-accidental deaths occurred during the months of June–August, including 14,779 among women and 13,358 among men. The average number of daily deaths was 38.70, with a minimum of 16 deaths per day, and a maximum of 73 for the whole period. During this interval, 75 heat-wave days occurred, including 39 before the HAP and 36 after.

Table 1 presents descriptive statistics before and after HAP implementation for non-eligible and eligible groups. The number of eligible hot days was similar between the two periods (39 and 36 days, respectively). Among eligible and non-eligible days, we did not find a difference in maximum temperature distribution between before- and after-program periods (Table 1). We found no difference in mortality distribution among non-eligible days before and after HAP implementation.

**Table 1:** Descriptive statistics before and after heat action plan program implementation for non-eligible and eligible days.

Variables^*a*^	Before program (2000–2003)		After program (2004–2007)	
	Non eligible group *n* = 329 days	Eligible group *n* = 39 days	Non eligible group *n* = 332 days	Eligible group *n* = 36 days
Median maximum temperature [°C (IQR)]	25 (22, 27)	32 (31, 33)	25 (22, 27)	31 (30, 32)
Number of hot days (%)	—	39 (11)	—	36 (10)
Daily number of deaths (IQR)	39 (36, 44)	44 (39, 52)	36 (32, 41)	39 (33, 43)
Daily number of deaths among men (IQR)	19 (16, 22)	20 (17, 24)	17 (14, 20)	18 (15, 22)
Daily number of deaths among women (IQR)	20 (18, 24)	24 (20, 29)	19 (16, 22)	21 (17, 25)
Daily number of deaths among elderly (≥ 65 years) (IQR)	32 (27, 35)	37 (32, 42)	29 (24, 32)	33 (28, 35)
Daily number of deaths among non-elderly (< 65 years) (IQR)	7 (5, 9)	7 (4, 9)	7 (5, 8)	6 (5, 8)
Daily number of deaths among low-SES group (IQR)	16 (14, 19)	20 (16, 22)	15 (12, 17)	16 (13, 18)
Daily number of deaths among high-SES group (IQR)	12 (10, 14)	13 (9, 16)	11 (9, 13)	12 (10, 14.5)
Daily number of daily differences in mortality according to sex^*b*^ (IQR)	2 (–2, 6)	4 (–1, 8)	2 (–2, 6)	4 (–2, 7)
Daily number of daily differences in mortality according to age^*c*^ (IQR)	24 (19, 28)	30 (26, 35)	21 (18, 26)	25.5 (20, 30)
Daily number of daily differences in mortality according to SES^*d*^ (IQR)	4 (1, 8)	6 (1, 12)	4 (1, 7)	3 (–1, 6)
^***a***^To compare maximum temperature distributions among eligible and non-eligible days between the two periods, we conducted a Kolmogorov–Smirnov test for equality of distribution functions. This test confirmed that there was no strong evidence of a systematic difference between the distribution of daily temperatures between the two periods (*p*-value > 0.10). For each continuous variable, mean values are presented followed by their IQR. ^***b***^Daily number of deaths among men – daily number of deaths among women. ^***c***^Daily number of deaths among elderly (≥ 65 years) – daily number of deaths among non elderly (< 65 years). ^***d***^Daily number of deaths among low-SES group (lowest tertile) – daily number of deaths among high-SES group (highest tertile).				

### Estimated Effect of HAP on Mortality

The adjusted DID model estimated that over the entire population, the HAP program reduced mortality during hot days by 2.52 deaths per day (95% CI: –0.34, 5.38). This result suggests that HAP may have helped reduce mortality during hot days.

### Estimated Effect of HAP on Equity

Adjusted estimates for the heterogeneous effect by sex, age, and SES are presented in Table 2. We estimated that HAP reduced differences in mortality between the elderly (≥ 65 years) and non-elderly (0–64 years) during hot days by 2.44 deaths per day (95% CI: 0.27, 4.59). We also estimated that HAP program reduced differences in mortality between individuals living in neighborhoods with low SES and those living in neighborhood with high SES (the middle tertile of SES was not included in the analysis) during hot days by 2.48 deaths per day (95% CI: 0.69, 4.27). However we did not find much evidence of heterogeneity in the program effect according to sex, with a DIDID estimate of 1.38 (95% CI: –1.60, 4.36).

**Table 2:** Estimated effect of the heat action plan program on equity.

Potential modifiers of the program benefits	Heterogeneity in the program effect^*a*^ estimate	95% CI	*p*-Value^*b*^
Sex (men vs. women)	1.38	(–1.60, 4.36)	0.36
Age (≥ 65 vs. < 65 years)	2.44	(0.27, 4.59)	0.03
Neighborhood SES (lowest SES tercile vs. highest SES tercile)	2.48	(0.69, 4.27)	< 0.01
^***a***^From DIDID (differences-in-differences-in-differences) estimates (Poisson model adjusted for temporal trends); 95% CIs were obtained by bootstrapping (1,000 samples). ^***b***^*p*-Values are obtained from a Wald test on the interaction term (i.e., DID estimate considering as health outcome the daily difference between two groups).			

### Sensitivity Analyses

Table 3 presents the adjusted DID estimates in sensitivity analyses. When we compared 4-year periods that were unrelated to HAP implementation, we did not find evidence of a difference in the effect of hot days on mortality between the earlier and later time periods based on the heterogeneity test. We also failed to find evidence of an effect on mortality when hot days were defined at a threshold of 28°C, thus misclassifying some days during which the Montreal HAP was not active as “eligible,” and when the threshold was 32°C, thus misclassifying some days when the HAP was active as “non-eligible. The latter result may be explained in part by the small number of hot days when using this definition (26 eligible days for the whole period). We then defined hot days according to cumulative heat (temperature on the day of death and up to the 5 days before death), and found that HAP program was effective in reducing daily mortality during hot days by 4.87 deaths per day (95% CI: 0.67, 8.20), a stronger effect, in comparison with 2.52 deaths per day (95% CI: –0.34, 5.38). Including a harvesting effect yielded a DID estimate of 1.87 (95% CI: 0.29, 3.47). Finally, restricting non-eligible days to maximum daily temperatures above 25°C yielded a DID estimate of 2.23 (95% CI: –0.80, 5.27), which is close to the point estimate of the main analysis but with a wider confidence interval.

**Table 3:** Sensitivity analyses for the estimated effects of the heat action plan program.

Sensitivity analyses	DID estimate	95% CI	*p*-Value^*a*^
Arbitrary programs
Program implemented in 2000^*b*^	0.94	(–2.08, 3.96)	0.54
Program implemented in 2002^*c*^	0.42	(–3.62, 2.77)	0.80
Other hot days definitions
When maximum temperature is above 28°C	0.58	(–1.77, 2.93)	0.63
When maximum temperature is above 32°C	2.79	(–2.65, 8.23)	0.32
Cumulative heat^*d*^	4.87	(0.67, 8.20)	0.03
Accounting for displacement ratio^*e*^	1.87	(0.29, 3.47)	0.02
Restriction to non-eligible days above 25°C	2.23	(–0.80, 5.27)	0.15
^***a***^*p*-Values are obtained from a Wald test on the interaction term (i.e., DID estimate). ^***b***^Using mortality and temperature data for periods 1996–1999 vs. 2000–2003. ^***c***^Using mortality and temperature data for periods 1998–2001 vs. 2002–2005. ^***d***^Considering a cumulative heat effect up to 5 consecutive hot days (lag 0–5). ^***e***^The displacement ratio (Saha et al. 2014) was 0.65.			

## Discussion

We found some evidence that the heat action plan implemented in Montreal in 2004 contributed to reduce mortality overall on hot days between 2004 and 2007. Furthermore, we observed that this program may have a positive effect in reducing some inequities in heat-related health impacts, because the estimated beneficial effects associated with the HAP were greater for elderly people and people living in low-SES neighborhoods. These findings show promise for further implementation of policies aimed at reducing the impact of extreme temperatures. Indeed, providing evidence about public health policies’ effectiveness may be useful to justify such investments for stakeholders/political leaders, and inform other jurisdictions that are considering implementing such policies. In addition, we apply this approach to policy impact evaluation.

To our knowledge, this is the first study to use a quasi-experimental method to estimate the causal effects associated with a HAP. In addition to providing relevant findings regarding this policy’s effectiveness in protecting populations, we feel that the quasi-experimental methodological approach is a major contribution to environmental health research. The DID approach that we used allows researchers to estimate a policy’s causal effect by using non-eligible days within the jurisdiction of interest, obviating the need for data from other cities or countries as control groups, the approach used in time series analyses with quasi-experimental methods (Cook et al. 2008). Our approach is distinct from previously published methods (Chau et al. 2009; Ebi et al. 2004; Fouillet et al. 2008; Morabito et al. 2012; Palecki et al. 2001; Tan et al. 2007; Weisskopf et al. 2002) because it controls for unmeasured confounders if the assumptions of the model are satisfied. For the DID method, the most important assumptions are the adequacy of the control group for the counterfactual contrast of interest and the absence of other interventions contemporaneous with the implementation of the new policy. In our setting where the control group was based on non-hot days in the same city, the validity of the causal estimates requires that *a*) there be no important time trends in the outcome of interest in the non-eligible group before and after policy implementation; and *b*) the distribution of the variable that defines the eligibility (temperature in this case) should not be different between the before and after periods.

Quasi-experimental methods have been widely used in recent years as alternatives to experimental methods and to observational designs that are affected by unmeasured confounders. They are designed to be more robust than pre–post comparisons that lack a control group (Angrist and Pischke 2008; Basu et al. 2016). In addition, the DID approach used here is not necessarily more difficult to implement than methods used previously (pre–post approach with a control group), provided that DID assumptions are met. In addition, our approach can be applied to other policies in which implementation is based on a daily threshold. For example, policies aimed at reducing air pollution levels on “smog episode days” (Zivin and Neidell 2009) (e.g., adjusting speed limits, alternative-day driving) can be evaluated following the same approach.

We also proposed a DIDID approach to evaluate heterogeneity in the HAP effect, and its potential impact on health equity. Our results suggest that targeting specific populations vulnerable to extreme heat (or other hazards) may reduce health inequalities between vulnerable and comparison groups. This might be explained by various actions undertaken as part of the HAP (Appendix 1) targeting directly some populations identified as vulnerable, such as frequent visits to home care patients (including elderly individuals) or daily phone calls to home care patients. DIDID have been used in studies with other exposures and health outcomes (Currie et al. 2014; Harper et al. 2014) to provide information about heterogeneity in policy effects. With this method, we propose a complementary approach that can be applied to policy evaluation using a time-series design either with or without a control group, as long as the data meet the assumptions described above.

This study is particularly relevant considering recent recommendations noted (Woodward et al. 2014) on the need for evidence of effectiveness of HAPs in promoting health and reducing health inequalities. Many countries, regions, and cities have recently instituted HAPs to reduce heat-related mortality and morbidity (Lowe et al. 2011; Toloo et al. 2013); however, few have evaluated the effects associated with their implementation. We therefore encourage further studies to estimate the effect of HAPs on mortality in other contexts. Further, these methods can also be used to assess effects on other health outcomes, such as hospitalization for fluid and electrolyte disorders or heat stroke (Bobb et al. 2014).

There are some limitations to our study. First, we assessed the effect of Montreal’s HAP without considering the spatial variability of benefits. Other studies have shown intra-city variability in health impacts of extreme heat (Hondula and Barnett 2014), and it is plausible that HAP benefits had a spatial pattern in our case as well. Further studies could conduct spatiotemporal analysis to address this gap. Because HAP measures have been triggered based on forecast daily temperatures, it is possible that some observed daily temperatures did not correlate with forecast data (Åström et al. 2014). In addition, by measuring temperature at a single site (i.e., the airport), we were not able to get within-city temperature variability that would inform us about exposure misclassification that might be correlated with neighborhood SES spatial variability (through micro–heat islands, for example). Another issue is redundancy between categories in the equity assessment (for example, prevented deaths among older individuals might be also counted in prevented deaths among individuals living in low-SES neighborhoods). We also did not distinguish actions undertaken > 30°C. Moreover, the Montreal HAP has been revised and updated yearly since 2007 (Price et al. 2013); this was not specifically considered in the present study. Further research could consider how incremental changes in policies affected mortality reduction, if at all. In addition, we did not qualitatively or quantitatively explore mechanisms through which the program contributed to reducing mortality during hot days. Implementation evaluation studies are a necessary complement to studies such as ours in producing a complete portrait of the effectiveness of a complex program such as a HAP.

Evidence of the effectiveness of HAPs is timely because the implementation of such programs has increased significantly across the world in recent years. Here, we provide evidence that a local program and a quasi-experimental methodology can help facilitate similar evaluations in other contexts.

**Appendix 1: Examples of Actions Pertaining to the Montreal Heat Action Plan**

Transmission of information coming from the Montreal PHD concerning levels of the plan that must be implemented in the health care network.
Intensification of surveillance of signs and symptoms of heat-related illness, reminder of preventive measures to patients, distribution of water bottles.
Air conditioning of common areas and opening of these areas during the day, the evening, and the night for patients in institutions.
Frequent visits to home care patients.
In institutions, frequent visits to housed patients.
Distribution of water, refreshments, lighter meals.
Monitoring of temperature in work areas, especially in warmer environments (e.g., kitchen, laundry room).
Frequent work breaks for workers in hot, non-air-conditioned environments.
Transfer of patients to common areas with air conditioning.
Transfer of vulnerable home care patients to air-conditioned shelters.
Daily contact by telephone or home visits to home care patients. Registry of calls and compilation of questionnaires for home evaluation.
